# Reference signal extraction from corrupted ECG using wavelet decomposition for MRI sequence triggering: application to small animals

**DOI:** 10.1186/1475-925X-5-11

**Published:** 2006-02-20

**Authors:** Dima Abi-Abdallah, Eric Chauvet, Latifa Bouchet-Fakri, Alain Bataillard, André Briguet, Odette Fokapu

**Affiliations:** 1 Laboratoire de Biomécanique et Génie Biomédical, UMR CNRS 6600, Université de Technologie de Compiègne, France; 2 Université de Picardie Jules-Verne, IUT de L'Aisne, France; 3 Laboratoire de RMN Méthodologie et Instrumentation en Biophysique, UMR CNRS 5012, UCB Lyon 1-ESCPE, France; 4 FRE 2678, Physiologie et Pharmacie Clinique, Université Lyon 1, France

## Abstract

**Background:**

Present developments in Nuclear Magnetic Resonance (NMR) imaging techniques strive for improved spatial and temporal resolution performances. However, trying to achieve the shortest gradient rising time with high intensity gradients has its drawbacks: It generates high amplitude noises that get superimposed on the simultaneously recorded electrophysiological signals, needed to synchronize moving organ images. Consequently, new strategies have to be developed for processing these collected signals during Magnetic Resonance Imaging (MRI) examinations. The aim of this work is to extract an efficient reference signal, from an electrocardiogram (ECG) that was contaminated by the NMR artefacts. This may be used for image triggering and/or cardiac rhythm monitoring.

**Methods:**

Our method, based on sub-band decomposition using wavelet filters, is tested on various ECG signals recorded during three imaging sequences: Gradient Echo (GE), Fast Spin Echo (FSE) and Inversion Recovery with Spin Echo (IRSE). In order to define the most adapted wavelet functions to use according to the excitation protocols, noise generated by each imaging sequence is recorded and analysed. After exploring noise models along with information found in the literature, a group of 14 wavelets, members of three families (Daubechies, Coiflets, Symlets), is selected for the study. The extraction process is carried out by decomposing the contaminated ECG signals into 8 scales using a given wavelet function, then combining the sub-bands necessary for cardiac synchronization, i.e. those containing the essential part of the QRS energy, to construct a reference signal.

**Results:**

The efficiency of the presented method has been tested on a group of quite representative signals containing: highly contaminated (mean SNR<-5 dB) simulated ECGs that replicate normal and pathological human heart beats, as well as some pathological and healthy rodents' actual ECG records. Despite the weak SNR of the contaminated ECG, the performances were quite satisfactory. When comparing the wavelet performances, one may notice that for a given sequence, some wavelets are more efficient for processing than others; for GE, FSE and IRSE sequence, good synchronisation condition is accomplished with coif5, sym8, and sym4 respectively.

**Conclusion:**

Sub-band decomposition proved to be very suitable for extracting a reference signal from a corrupted ECG for MRI triggering. An appropriate choice of the wavelet function, in accordance with the image sequence type, could considerably improve the quality of the reference signal for better image synchronization.

## Background

Magnetic Resonance Imaging (MRI) has become by far the primary tool for gaining important insights into the functional and metabolic bases of heart disease. However, the observation of a moving organ, such as a beating heart, requires synchronization: since an image cannot be acquired in one heart cycle, its successive acquisitions have to be accurately combined with the cardiac phase motion. Such requirements depend on a reliable detection of the R-wave of the electrocardiogram (ECG) to guarantee that consecutive image data collections always start at the same point of the cardiac cycle. Still, a fundamental problem for monitoring a subject's cardiac activity during MRI is the corruption of the ECG signal due to adverse electromagnetic effects [1-4]. The oscillating magnetic fields may induce voltage artefacts which do not reflect actual electrophysiological events. This effect is particularly pronounced in small animals MRI microscopy, where strong and rapidly-switching gradients, leading to elevated induced voltages, are needed to obtain high spatial and temporal resolution. Conversely, a small animal's ECG is just few millivolts in amplitude and with the spurious signals often resembling the QRS spike, correct cardiac gating is often greatly disrupted. Recent works have proposed to improve motion gating strategies [5,6], however the system presently commercialized for small animal monitoring is still in its early development stage.

During cardiac MRI, the signal acquired by the ECG sensor does not only contain the electrophysiological information, it also includes some components generated by the NMR environment, and the collected signal S(t) can be modelled as [3]:

S(t) = S_el_(t) + S_flow_(t) + S_move_(t) + S_MR_(t) + S_rf_(t)     (1)

S_el_(t) represents the signal to be analysed (ECG). S_flow_(t) is induced by the magnetohydrodynamic effect, and S_move_(t) is due to subject-related sensor motions in the magnetic field (respiration, heart beat and voluntary motion). These two contaminating signals cover a range of several Hz which may overlap the ECG spectrum. Radiofrequency pulses are of several MHz (typically 64 MHz at 1.5 T) and induce the S_rf_(t) noise in this frequency range or in the order of the inverse of duration pulses, which is several tens of kilohertz. S_MR_(t) is due to the temporal variations of the magnetic field gradients which are typically switched during some 10 ms, thus the corresponding induced voltages are in a frequency range of several Hz to kHz. The frequency distribution of the overall signal is a major point to take into account if any filtering is to be carried out, since the artefacts to be removed should be distinguishable from the desired signal (ECG) which is at several Hz. But given that there is some spectral overlapping between the noise and the desired components of the ECG, we turned to wavelet decomposition which has proven to be particularly well suited for such cases [7-10]. ECG signals are characterized by a cyclic occurrence of patterns (QRS complexes, P and T waves) with different frequency content. Power spectral analysis of the ECG shows that, P and T waves usually have an important spectral density up to 10 Hz only, while most of the QRS power lies in the 3–17 Hz band [11]. Moreover, NMR induced noise and artefacts disturbing the ECG signal appear at disparate frequency bands. Thus, a strategical approach for detecting heartbeats is to analyze the different sub-bands of the ECG [12], for this we make use of the wavelet transform that can provide a description of the signal in the time-scale domain, allowing the representation of the temporal features of a signal at different resolutions. By exploiting this powerful tool here, we aim to extract a reference signal from a contaminated ECG signal. This reference signal can be either used for subject heart rate monitoring or for synchronization during MRI scans. The proposed extraction method relies on a multidimensional modeling technique and it consists in decomposing the contaminated ECG signal into frequency sub-bands using the wavelet transform. Since the wavelet shape must be carefully adapted to achieve good event detection, the choice of the wavelet function is at the center of this study. The wavelets already used to detect QRS complex are considered again. Furthermore, some particular characteristics of artefact noise generated by the NMR environment are also taken into account. The extraction procedure is applied to several signals using various wavelet functions, to determine the most suitable wavelets to locate R peaks according to the employed MRI sequence.

## Methods

### Experimental setup and data acquisition

The experimental setup involves a 2 Tesla OXFORD 85/310 horizontal cryomagnet equipped with a 50 mT/m gradient system, and a MR compatible ECG sensor which technical details are provided in [1]. This device is designed to reduce the interaction sources: three bypasses (100 pF capacitors) guarantee a reduction of RF contamination of the ECG amplifier by the MR system. The ECG is amplified and converted into an optical signal which is transmitted out of the magnet bore by optical fibre and then converted back to electrical. The experimental signals were detected using three carbon electrodes (3M™ Red Dot™ Radiolucent Electrode), then low-pass filtered (0.5 -20 Hz) and sampled at 1 kHz. A digital signal processing system (NI DAQCard-6024E, and a PC with Matlab) was placed outside the Faraday cage for data acquisition, processing and storage.

Ten seconds of signal were recorded during MR scanning using three imaging sequences: Gradient Echo (GE), Fast Spin Echo (FSE) and Inversion Recovery with Spin Echo (IRSE). The scan parameters for theses sequences are outlined in Table 1 and they correspond to mice cardiac MRI parameters.  Three types of data were recorded:

**Table 1:** Description of rodents and imaging sequence parameters

	Weight	Sequence parameters (TR/TE in ms)		
		GE	FSE	IRSE

rat	170 g – 550 g	150/5	1700/18	1900/30
mouse	30 – 32 g	385/10	370/10	370/10

**Figure 1:**
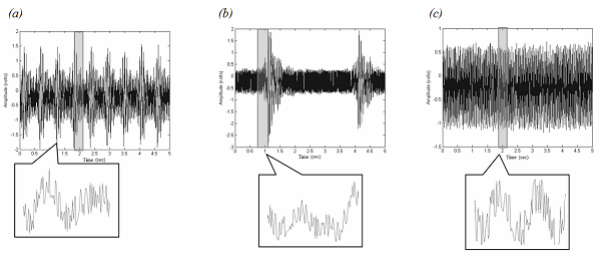
**Recorded noise signals. **Zoomed portions of the recorded noise during each of the three imaging sequences show various shapes. (a) IRSE noise (b) FSE noise, and (c) GE noise

*a) Noise generated by the NMR environment: *two electrodes, immersed in a conducting fluid (salt water) made it possible to collect noise signals corresponding to three NMR sequences (figure 1). These signals, mainly due to gradient artefacts, would then be analysed to select the wavelets for ECG decomposition.

**Figure 2:**
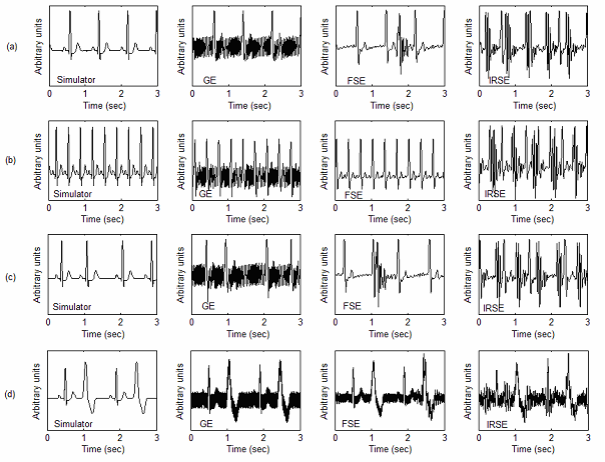
**Simulated ECG recorded before and after contamination. **(a)75 bpm (b)180 bpm (c)Sinus Arrythmia (d) Bigeminy.

b) *Corrupted simulated ECG: *an ECG simulator (phantom 320 GmbH Mebsystem für Medizintechnik) placed outside the magnet provided signals that were driven inside the MRI tunnel using carbon cables. These signals could then be contaminated by the NMR artefacts, while the imaging sequences were activated. The simulated signals included 4 ECG types: *Two Normal simulated ECGs *denoted "75 bpm" (simulation of a normal human ECG) and "180 bpm" (simulation of a normal small animal ECG: high frequency heart beat) where bpm represents the beat number per minute. *Two Pathological simulated ECGs *denoted "Bigeminy" (simulation of premature ventricular contraction where an elevated ventricular premature beat follows each normal beat) and "Sinus Arrhythmia" (simulation of variable R wave occurence periods). The choice of the ECG signal types was directly related to the study context, especially in the case of pathological signals that involve rather distorted characteristics. Figure 2 shows the four simulated ECGs before (recorded outside the NMR environment) and after (recorded in the magnet bore) contamination by the noise of the different sequences. For each signal, the contamination level was evaluated, according to the sequence noise, resulting in the following SNR mean values: -5.92 dB for GE, -5.07 dB for FSE, and -8.15 dB for IRSE. Such poor SNR are due to the long cables necessary to drive the signal from the simulator device to the MRI magnet bore. They constitute after all, a very interesting aspect for evaluating the effectiveness of our algorithm.

*c) Rodent bipolar ECG signals: *They were collected, during MR scanning, on anaesthetized mice and rats which weight ranges are given in Table 1. Rodents were anesthetized by intraperitoneal injection of sodium pentobarbital (50 mg/kg, Sanofi, France).  ECG electrodes were positioned on the animal's thorax in a lead II configuration. The ECG sensor was placed close to the animal, and short cables were used to connect it to the electrodes, thus avoiding additional artefacts. The animal was then secured on a cradle, inside a birdcage RF coil, and placed at the centre of the gradient set.

### Selection of the wavelet functions

There is no absolute rule to determine the most adequate analyzing wavelets; the choice must always be specific to the application as well as to the analysis requirements [13]. Since the characteristics of the noise generated by the NMR environment vary greatly according to the excitation sequence, one may have to test a large number of wavelets in order to associate the most appropriate wavelet with each imaging sequence. For this study, three wavelet families were retained that represent the most commonly used families of orthogonal wavelets for detecting ECG events: Daubechies, Coiflets and Symlets. The members of the corresponding families to use were then picked out: on the one hand by relying on a thorough investigation of related literature. And on the other, by analysing noise characteristics observed during the different excitation sequences.

#### a) Wavelet selection based on literature data

Among the wavelet families mentioned above, we selected the members that have proven to be very efficient for ECG denoising and/or QRS complex detection. Some of the wavelets used in compression techniques, where the preservation of the ECG waveform morphology is of great importance, were also retained. The Daubechies wavelets have shown to be very adequate for noise reduction [14,15], baseline wandering removal [16], and QRS detection [17], they are also widely used for ECG compression [18-21]. Such applications have usually required the use of db1, db3, db4 or db6. On the other hand, due to their redundancy, the Coiflets insure minimum signal degradation and provide a convenient technique for QRS extraction [19], and data compression [22]. In particular, coif2 and coif3 were used for cardiac arrhythmia classification algorithms [23,24]. Conversely, the Symlets were mainly chosen for their resemblance to the ECG signal shape [25]. Their efficiency has been reported for ECG denoising and compression, particularly the sym3, sym 4 and sym6 wavelets [19,20]. According to this bibliographical report, the first group of wavelets to be tested by the proposed algorithm was composed of: db1, db3, db4, db6, coif2, coif3, sym3, and sym4.

#### b) NMR noise resembling wavelets

**Figure 3:**
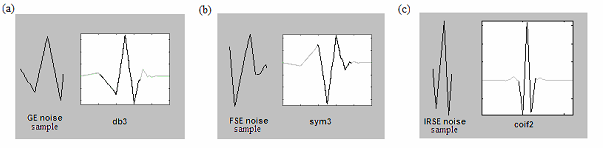
**Example of comparison between noise shapes and some wavelets. **FSE, GE and IRSE sequences, which resemble the db3, sym3 and coif2 wavelets respectively.

For this second selection, the investigation was enlarged to include wavelets generally used for denoising or detecting events in various electrophysiological signals (ElectroCardioGram, ElectroEncephaloGram, ElectroMyoGram, ElectroOculoGram) We set aside the wavelets, members of the three previously mentioned families, which have been used for applications such as EEG, EOG and uterine EMG denoising [26-30], respiration and arterial pressure multiresolution analysis [26], foetal ECG extraction [31], analysis of time-frequency characteristics of motor unit action potentials [32], and EMG decomposition [33,34]. The gathered wavelets were then compared with the most representative samples (temporal motifs reproduction) of the noise signals, recorded during the three imaging sequences. The most distinguishable noise samples showed resemblance to db3, sym3, coif2, sym4, sym5, sym7 wavelets; an illustration is given figure 3. It was also possible to depict samples similar in shape to db4, coif3, sym3, and sym6. To this wavelet group we added coif4 and coif5 which resemble coif3, as well as sym8 which looks like sym6. By putting together those noise resembling wavelets, with the set composed in (a) we get a group of 14 wavelets to be tested with the elaborated algorithm: *{db1, db3, db4, db6, coif2, coif3, coif4, coif5, sym3, sym4, sym5, sym6, sym7, sym8}*.

### The algorithm

**Figure 4:**
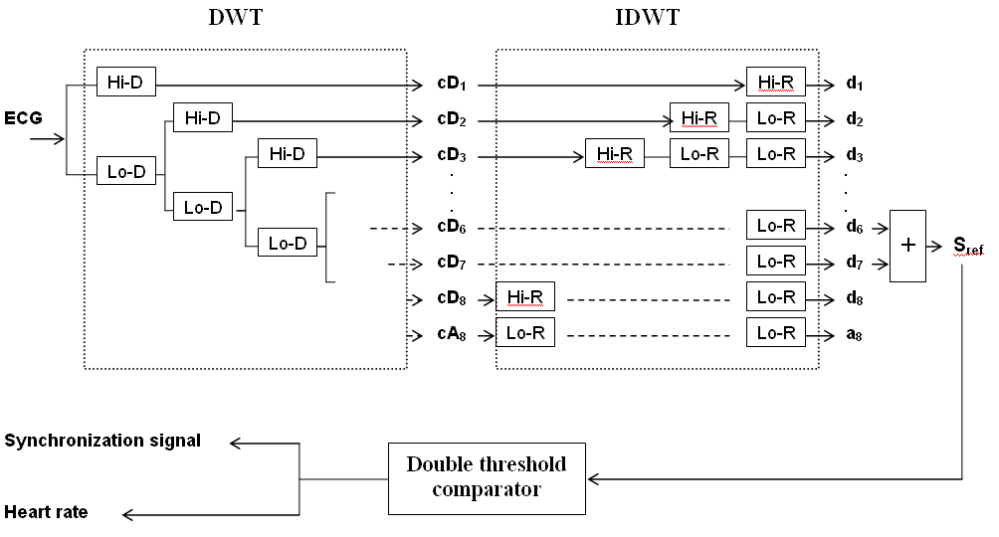
**Overview of the extraction algorithm. **The contaminated ECG is decomposed into 8 scales; the reference signal is then constructed by adding together the 6th and 7th sub-bands signals. A simple trigger generates the cardiac rhythm as well as the synchronization signal. Hi-D and Hi-R are respectively the high pass decomposition filter and its associated reconstruction filter. Lo-D and Lo-R represent the low pass decomposition and reconstruction filters. cD_j _denotes the detail coefficients, and d_j _are the reconstructed detail signals.

The algorithm (figure 4) includes three main steps:

i) Frequency sub-band decomposition of the contaminated ECG signal based on discrete wavelet transform.

ii) Reference signal construction by merging the detail signals where the QRS complexes have the most important frequency components.

iii) Trigger extraction by applying a threshold to the reference signal.

#### Frequency sub-band decomposition of the contaminated ECG

The sub-band decomposition is based on the orthogonal discrete wavelet transform (DWT). Detailed description of the theory and implementation of DWT is given in [35]. To summarize, the decomposition of the signal with DWT is based on a partition in the frequency plane using a quadrature mirror filter (QMF) bank [36]. The filter bank is made up of pairs of digital high-pass and low-pass filters organized in a tree structure. At each level, the signal is decomposed into detail (high frequency components) and approximation (low frequency components) coefficients and is then down-sampled. The detail coefficients are afterwards stored and the decomposition continues by filtering the obtained approximation. At each level j, the frequency axis is recursively divided into halves at the ideal cut-off frequencies.

f_j _= 2^-j^.1/2T (where T is the sampling period)     (2)

The number of decomposition scales in this work was determined referring to [11] where it was shown that most of the QRS energy lies in the 4–17 Hz band. Accordingly, the choice of the number of decomposition scales should be performed in such a way as to isolate this particular bandwidth. Thus, the contaminated ECG signals sampled at 1 kHz were decomposed into 8 frequency sub-bands resulting in the following filter banks bandwidth: 1.96, 3.91, 7.81, 15.63, 31.25, 62.5, 125, and 250 Hz. For each level, detail signals were then reconstructed, using low-pass and high-pass reconstruction filters with up-sampling.

#### Extraction of the reference signal

Here 'reference signal', denoted as S_ref _represents an ECG model where the QRS features have the major frequency components. After reconstruction of the detail signals, the reference signal could be performed by summing details d_6 _and d_7 _resulting in a [3.91–15.63] Hz sub-band.

#### Trigger computation

The computation is based on a simple Schmitt trigger principle. A double threshold comparator is applied on S_ref _: It switches the output to a high state when the input passes upward through a high threshold value ***ht***. It then prevents switching back to the other state until the input passes through a lower threshold value ***lt***. The comparator thus produces a TTL signal that reflects the R wave recurrences and can be directly used for sequence triggering and for mean heart rate estimation. Note that the high and low threshold values are defined as a percentage of the S_ref _amplitude maximum and can be adjustable by the experimenter via a graphical interface.

### Evaluation criteria

In order to achieve a quantitative evaluation of the algorithm performances for each wavelet, two parameters, usually employed to evaluate detection algorithms, were computed. The sensitivity (Se) and the positive predictive value (+P) of the present QRS detection algorithm were defined as:





where TP is the number of true positives, FN the number of false negatives, and FP the number of false positives. The sensitivity Se represents the percentage among real beats of those that were correctly detected by the algorithm. The positive predictive value +P reports the percentage among beat detections of those corresponding to real beats. These two parameters can be combined into one: the Diagnostic Quality Factor (DQF) given as the geometric mean of Se and P+. Furthermore, the mean DQF (mDQF) can be computed as the arithmetical mean of a set of DQF values:



However this only gives a rough estimate of the performances, since Se and P^+ ^contribute equally in the calculation, while in fact the weight of each should vary depending on the considered application. In the case of QRS detection for cardiac MRI synchronization, the number of FP is more crucial than the FN. In fact, FP detections deteriorate the image quality because acquisitions are triggered at inappropriate moments, blurring the image, whereas FN has no consequence on the image itself; the missed QRS complexes only extend the image acquisition duration. The ideal situation would be FP = FN = 0 (Se = +P = 100%) where the best image quality could be achieved in the shortest time. The purpose of this algorithm was to seek the wavelet that yields a FP value that tends to zero (+P tends to a 100%) with a minimum of FN (maximum Se) according to the applied MRI sequence.

## Results

The algorithm performances were evaluated for each one of the selected wavelets using both *real *and *simulated *ECG signals. The test results for each signal type according to the applied sequence and the analyzing wavelet is presented (Table 2). Wavelet performances appeared to vary with the signal type as well as with the noise nature (sequence type). The results were also averaged to attain a global view of the performances of the whole wavelet group according to every sequence (Table 3) as well as the performances of each individual wavelet regardless of the sequence type (Table 4).

**Table 2:** Performance results for each of the tested wavelets according to the applied imaging sequence

	**Simulated ECG**												**Rodents ECG**			
		
	GE				FSE				IRSE				IRSE			
		
	FP%	FN%	Se	+P	FP%	FN%	Se	+P	FP%	FN%	Se	+P	FP%	FN%	Se	+P
**db1**	0.00	1.67	98.33	100	2.33	8.54	91.46	97.47	1.67	57.48	42.52	93.33	0.00	9.46	90.54	100
**db3**	0.00	11.21	88.79	100	1.67	15.41	84.59	98.33	10.00	33.93	66.07	90.26	0.00	4.73	95.27	100
**db4**	0.00	4.87	95.13	100	0.67	7.00	93.00	99.13	12.33	38.03	61.97	87.00	0.00	2.70	97.30	100
**db6**	1.67	6.00	94.00	98.5	3.21	15.41	84.59	96.67	6.67	49.97	50.03	92.00	0.00	0.68	99.32	100
**sym3**	0.00	11.21	88.79	100	1.67	15.41	84.59	98.33	10.00	32.67	67.33	90.26	0.00	5.41	94.59	100
**sym4**	0.00	10.67	89.33	100	1.67	11.87	88.13	98.18	0.00	41.33	58.67	100	0.00	2.03	97.97	100
**sym5**	0.00	2.00	98.00	100	4.74	7.21	92.79	95.48	18.33	45.33	54.67	80.23	0.00	0.68	99.32	100
**sym6**	0.00	0.00	100	100	1.67	6.87	93.13	98.46	0.00	44.00	56.00	100	0.00	1.35	98.65	100
**sym7**	0.00	2.67	97.33	100	0.00	10.67	89.33	100	13.33	38.67	61.33	89.03	0.00	0.00	100	100
**sym8**	0.00	0.67	99.33	100	1.67	4.21	95.79	98.46	0.67	46.67	53.33	99.05	0.00	0.68	99.32	100
**coif2**	0.00	0.67	99.33	100	1.67	13.95	86.05	98.18	0.00	45.67	54.33	100	0.00	3.38	96.62	100
**coif3**	0.00	0.00	100	100	0.00	10.82	89.18	100	1.67	47.33	52.67	97.78	0.00	0.00	100	100
**coif4**	0.00	0.00	100	100	0.67	11.95	88.05	99.20	1.67	59.33	40.67	97.50	0.00	0.00	100	100
**coif5**	0.00	0.00	100	100	2.21	4.87	95.13	97.82	11.67	28.67	71.33	91.27	0.00	0.00	100	100

(All values are expressed in %)

FP %= FP/nQRS, FN%= FN/nQRS, nQRS = total number of QRS peaks in the signal

**Table 3:** Averaged results for each MRI sequences

*mDQF*	**Simulated**	**Rodent**
**GE**	98.06%	100.00%
**FSE**	93.86%	100.00%
**IRSE**	72.34%	98.87%

For each sequence the Diagnostic Quality Factor is averaged over 14 wavelets

**Table 4:** Averaged results for each wavelet

*mDQF*	**Simulated**	**Rodent**
**db1**	85.52%	98.38%
**db3**	87.55%	99.20%
**db4**	88.99%	99.55%
**db6**	84.82%	99.89%
**sym3**	87.80%	99.09%
**sym4**	88.04%	99.66%
**sym5**	86.45%	99.89%
**sym6**	90.20%	99.77%
**sym7**	89.02%	100.00%
**sym8**	89.82%	99.89%
**coif2**	88.43%	99.43%
**coif3**	88.73%	100.00%
**coif4**	85.48%	100.00%
**coif5**	92.38%	100.00%

For each wavelet the Diagnostic Quality Factor is averaged over 3 sequences

### Simulated signals

**Figure 5:**
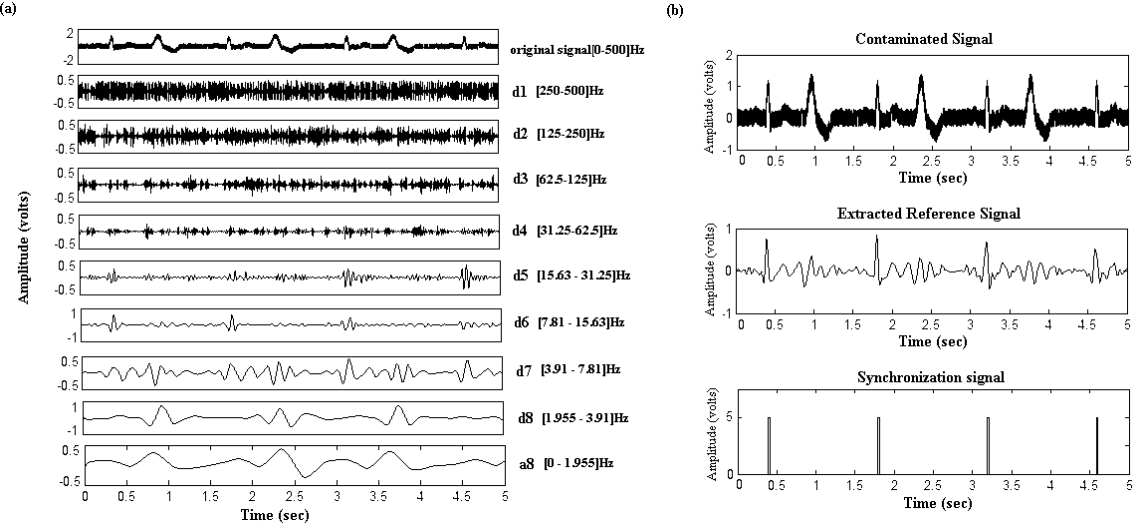
**Processing example of a simulated pathological ECG. **(a)Decomposition into sub-bands of a "Bigeminy" signal contaminated by the GE sequence noise (b) Extraction of the reference and synchronization signals.

**Figure 6:**
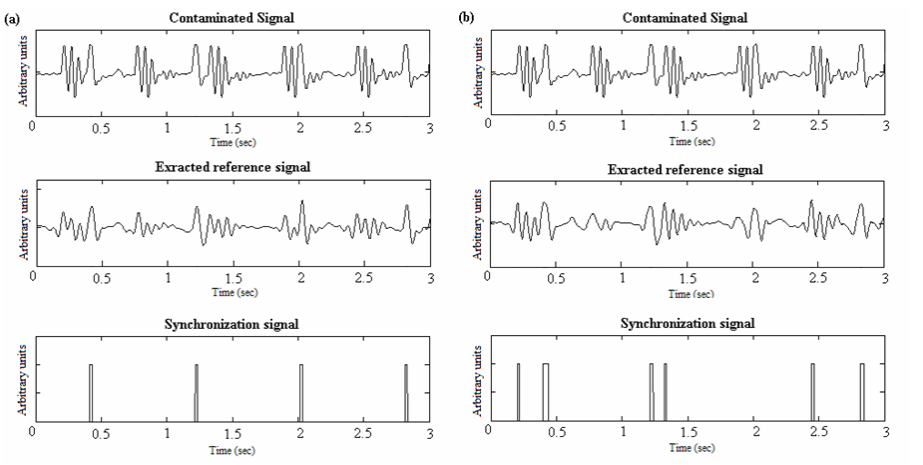
**Wavelets performances difference. **QRS detection for "75 bpm" signal recorded during the IRSE sequence: the same signal is decomposed using coif5 and sym5. One can clearly notice the superiority of coif5 (a) (that ensured perfect detection), to sym5 (b) (that detected 3 false peaks and missed one QRS).

A total of 12 simulated signals (4 signals for each of the three sequences) were processed with the proposed algorithm using each of the 14 analyzing wavelets. For the GE sequence, the algorithm achieved a perfect QRS detection for all ECGs. For FSE and IRSE sequences, we noticed that the heart beat frequency might affect the detection efficiency. In fact, when fast heart beating rates ("180 bpm") are involved, there is a higher probability that the noise generated during gradient activation time may coincide with the QRS complex, leading to confusion. On the other hand, the beat irregularity had no major effect on the performances. As for the "bigeminy" signals, the algorithm successfully distinguished between real QRS complexes and premature beats like illustrated in figure 5. In this example, premature beats are very prominent, but due to the fact that their energy contribution is weak in details 6 and 7, a correct QRS detection was obtained. Table 2 shows, for a given wavelet, and a given sequence, the evaluation criteria averaged on all 4 signal types. When comparing the wavelet performances according to the noise type, one may notice that for a given sequence, some wavelets are more efficient for processing than others (an example is given figure 6). If one considers that a proper synchronization condition is accomplished when +P presents a high value while FN is low, the following can be noted:

a) For a given wavelet, the best results are obtained for the GE sequence. For the FSE sequence, the algorithm is slightly less effective, but it is still better than the IRSE case.

b) The wavelet performances do not only depend on the noise level, but on its energy distribution as well. Despite the fact that the FSE signals have better SNR than the GE signals, the algorithm leads to lower error rates for all wavelets in the case of GE contamination. This can be explained by the fact that the noise generated by the GE sequence has relatively weak amplitude, spreading all over the signal duration; whereas the high amplitude FSE noise is concentrated in some localised parts of the signal. Which is why, despite its weaker energy, the FSE noise makes the QRS detection even harder, since its elevated peaks may be mistaken for QRS complexes.

Roughly one may conclude that it is the Coiflet family that achieves quite reliable detection. However, if we examine each sequence individually, we can define, for each, a group of the most efficient wavelets. For the GE sequence, almost all of the wavelets yielded good results. Nevertheless, it was the sym6, coif3, coif4 and coif5 which were able to achieve a perfect detection rate. As for FSE sequence signals, we can say that processing with db4, sym6, sym7, sym8 and coif3, led to the lowest error rates. Finally, for the IRSE, sym4, sym6 and coif2 proved the superiority of their performances.

### Rodent ECGs

**Figure 7:**
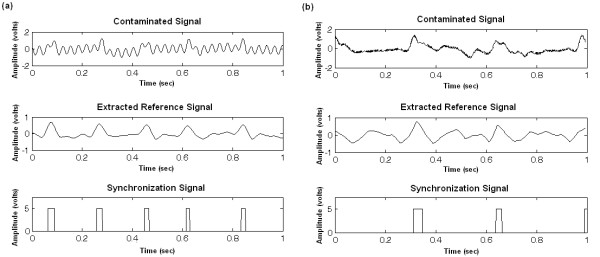
**QRS detection on contaminated rodent ECG. **(a) Mouse ECG recorded during an IRSE sequence and processed with coif3. Despite the low SNR and the very large base line drift, the algorithm succeeded in ensuring a perfect QRS detection. (b) ECG of a hypertensive rat with bundle branch block beat recorded during a FSE sequence, where one can clearly depict the effect of the pathology on the QRS morphology. After processing with the sym4 wavelet, the QRS are very well marked in the extracted reference signal.

As opposed to the simulated signals where the simulator had to be placed outside the MRI tunnel and long cables used to drive the signals inside to be contaminated, the rodent ECGs were detected on the animal placed directly inside the tunnel. With this setup, the rodent signals were less contaminated than the simulated ones. However, the difficulty of synchronization in this case is due to the animal's physiology resulting in weak ECG amplitude and very fast heart rate. Not to forget supplementary artefact occurrences such as respiration. Figure 7 shows two illustrations of synchronization signal extraction from an ECG of a healthy mouse and that of a hypertensive rat. The QRS detection results on the rodent ECGs are summarized in Table 2, they show that, for signals recorded during GE and FSE sequences an ideal case of accurate detection (Se = +P = 100%) was attained. For the IRSE sequence, which is the most contaminated, the detections were less efficient with, however, no false detection but a few missed peaks, therefore slightly extending the image acquisition duration. The wavelets that ensured perfect detection for this sequence were the sym7, coif3, coif4 and coif5.

## Discussion and conclusion

Since wavelet transform has long proven its efficiency for QRS detection in standard ECG signals, we developed a signal processing algorithm found on a wavelet-based filter bank decomposition strategy, that allows the extraction of an efficient reference signal from a contaminated ECG, mainly for MRI synchronization. Considering the technical aspects of the proposed algorithm, the following remarks can be made:

**(i) Selection of the wavelet functions : **The efficiency in extracting a given signal based on wavelet decomposition depends greatly on the choice of the wavelet function and on the number of decomposition scales [13]. In this study, a straightforward approach for wavelet selection was based on: 1°) Literature where wavelets have already been used for ECG processing. Three wavelet families Daubechies, Coiflets, Symlets were selected. 2°) Analysis of MRI sequence generated noise signals, based on the similarity between noise samples and some members of the three considered families. These two approaches resulted in an optimal group of 14 wavelets to be tested in order to define the most appropriate wavelets for each excitation sequence. Despite the fact that this selection method has produced efficient results, one may notice that it is more or less subjective.

**(ii)Elaboration of a reference signal : **the number of scales was fixed according to ECG spectral analysis, while considering the frequency components of the contaminating artefacts. The choice of the number and levels of the details for the reference signal reconstruction was guided by the work of Thakor et al. [11]. Only the sub-bands necessary for cardiac synchronization (those containing the essential part of the QRS energy) were taken into account: the complete reconstruction of the ECG signal is unnecessary for gating applications. For an ECG sampled at 1 KHz, the decomposition process over 8 levels perfectly isolated the QRS complexes (details d_6 _and d_7_) from the undesirable components of the contaminated ECG, such as the NMR environment artefacts (d_1 _to d_5_), the P and T wave (d_8_), the baseline drift and the respiration signal (a_8_). The reference signal extraction was thus performed by combining the scales spanning over the [3.91–15.63]Hz range.

**(iii) Algorithm performance : **the efficiency of the presented method was tested on a group of quite representative signals: 1°) highly contaminated (mean SNR<-5 dB) simulated ECGs of normal and pathological heart beats, which provided a very interesting aspect for the algorithm evaluation, given that the positions of the QRS in the signal were known *a priori *and hence could be compared to the result of the extraction algorithm. 2°) real ECG signals recorded on healthy and pathological rodents during cardiac MR imaging where the high and fast switching gradients as well as animal physiology make QRS detection rather difficult.

In order to quantitavely evaluate the algorithm, two coefficients (+P and Se) were calculated for each wavelet and each sequence. On the basis where the highest +P and lowest false detections are sought, the process turned out to be especially reliable for small animal signals. As for the simulated ECGs, despite their weak SNR, the performances were quite satisfactory for both GE and FSE sequences. However, the method was less efficient in the case of the IRSE sequence, where the positive predictive value was suitable, but the false negative detections rate remained too high (>30%). This is certainly due to the particularly unfavourable contamination conditions that led to a very low SNR. As a matter of fact, the use of long cables generates supplementary artefacts in the signal which are not observable in the case of real simultaneous ECG recordings. So even though the signals were recorded during basic sequences (GE, FSE, IRSE) which contaminate the ECG less than more complex ones, such as echo planar, the obtained noise level was somewhat comparable to that of sophisticated sequences.

After having computed the evaluation criteria for all signals, we tried to establish which were the most efficient wavelets for each sequence: For instance, all tested coif wavelets, achieved perfect detection for the GE sequence, whereas for the FSE, it was the sym8 that produced the lowest error rates and the sym4 that yielded the most acceptable results for the IRSE.

Globally, the results produced by the whole selected wavelets set were quite acceptable, for all three considered sequences. However, later applications of the method for very complex sequences might necessitate the use of other wavelets such as the biorthogonal ones, which have already been suggested for QRS detection in extreme conditions and for intensive care patients [37]. The algorithm has proven to be very efficient for small rodents' signals but further research is needed to investigate whether the obtained results could be reproduced in human subjects.

In conclusion the proposed wavelet-based technique has shown to be particularly efficient in extracting a good quality reference signal for synchronization during MRI examination. Given the fact that only the details essential for synchronisation are used for the signal reconstruction, both low and high frequency artefacts are correctly removed; the synchronization problem caused by baseline wandering is thus solved. The highest degree of accuracy of our algorithm was obtained when wavelet functions deduced from noise analysis were used to decompose the contaminated ECG. In an upcoming paper we intend to investigate the real-time implementation of the method, indispensable for the use in cardiac MRI applications.

## Authors' contributions

DA and OF conceived the method, implemented the algorithm and drafted the manuscript. EC and LB participated in the signal acquisitions. ABr carried out work coordination and manuscript revision. ABa provided pathological animal models. All authors read and approved the final manuscript.
